# Guilt and shame and its relation to oxytocin in patients with depression and alcohol addiction

**DOI:** 10.1186/s12888-025-06762-y

**Published:** 2025-04-02

**Authors:** Paraskevi Mavrogiorgou, Patric Dalhoff, Georg Juckel

**Affiliations:** Present Address: Department of Psychiatry, Psychotherapy and Preventive Medicine, LWL-University Hospital of Ruhr-University Bochum, Alexandrinenstr.1, Bochum, 44791 Germany

**Keywords:** Guilt, Shame, Depression, Aalcohol dependence, Oxytocin

## Abstract

**Backround:**

Guilt and shame are important and universal social emotions that fundamentally shape the way people interact with each other. Mental illness such as depressive disorder (DD) or alcohol addiction (AA) is therefore often related to pronounced dysfunctional feelings of shame and guilt. Oxytocin has been suggested to play an important role in socially and morally associated emotions such as shame and guilt.

**Methods:**

A total of 85 participants (41 women and 44 men) were clinically investigated, including shame and guilty proneness. To assess the proneness for guilt and shame, the IGQ, the SCV scale, TOSCA, and SHAME were used.

**Results:**

Patients with DD showed a maladaptive guilt and shame profile, characterized by increased interpersonal feelings of guilt and increased proneness of shame. Patients with AA were characterized by the lowest reserve and antidelophilic attitude. Oxytocin values were lowest in the patients with AA compared to the two other groups, but not related to guilt and shame.

**Conclusion:**

The proneness to maladaptive guilt and shame of mental disorders appears to be less dependent on specific disease aspects than on individual characteristics. Dimensions such as guilt and shame should be more implemented in psychotherapy.

## Background

Guilt and shame, along with pride and embarrassment, are among the self-evaluative emotions. They arise from discrepancies between our behavior or characteristics and our moral standards [1]. They are also considered as social emotions that assume and express presumed negative external evaluations of an individual’s own actions or behavior. Moreover, they are also an expression of the person's awareness of this evaluation e.g. in considering the own addiction or depression [2]. The social character of these two emotions is made clear by the fact that they occur primarily in interactions with other people. In everyday life, the main aim is to avoid these emotions, as they are often attributed as something unpleasant [3]. However, guilt and shame are important and universal social emotions that fundamentally shape the way people interact with each other [4]. For example, feelings of shame are attributed a relevance for interpersonal coexistence or protection of self-integrity [5]. Feelings of guilt can strengthen feelings of community, as both emotions, when expressed appropriately, have a protective and relationship-regulating character. For a long time, guilt and shame were regarded as two different expressions of the same emotion [3]. Differentiation is difficult, not least because the two emotions are often used synonymously and yet each of the two emotions can also merge into the other [5]. A first attempt at differentiation is that of H.B. Lewis in the 1970s, who understood guilt as an explicit act, action, or omission, whereas according to her thesis, shame would refer to the evaluation of the whole self, thus to the person and not to a specific action [6]. It was found that there does not necessarily have to be a public audience for the feeling of shame to be prevalent [7].

In the literature, shame is usually associated with negative self-judgement and self-contempt [8, 9]. Shame is also often attributed more negatively and perceived as more difficult to bear than guilt. With both emotions, however, if the emotional reaction exceeds what is appropriate, maladaptivity or dysfunctionality can result [4]. This is determined by the occurrence of intensified and generalized guilt and emotions of shame. Feeling of shame is also characterized by the fact that the emotionally experienced sense of self-worth of the affected person is perceived as deficient [10]. In summary, both emotions, i.e. guilt and shame, can cause intrapsychic pain [11]. Mental illness is therefore often related to pronounced dysfunctional feelings of shame and guilt. An increased sense of shame is reported, for example, in addiction disorders [12, 13]. Here, shame refers on the one hand to self-deprecation of one’s own person, associated with anxious-depressive psychopathology, which is to be denied with the help of a drug [14]. On the other hand, so-called secondary shame occurs in the context of the addiction disease, as the person affected is usually aware of their addiction, but at the same time tries to deny this. This fact is often perceived as shame and stigma and leads to hidden addictive conduct (i.e. clandestine drinking behavior). Those affected often feel great shame afterwards that other people have witnessed their social aberrations and addiction [5, 12, 13]. A close association of guilt and shame is found in the literature, especially in depressive disorders [15, 16]. Feelings of shame and guilt are mixed, and the depressed person practices self-blame and feels guilty because of their perception of the failure of others [5]. Based on a few empirical studies, guilt and shame as essential social emotions seem to play an important psychopathological role in the development of mental disorders.

According to our current knowledge, there have been no neuroendocrinological studies on the development and expression of feelings of shame and guilt. Although the endogenous hormone oxytocin is considered to play a special role in socially and morally associated emotions, to which feelings of shame and guilt can also be counted [17], there are only few neurobiological studies on this [18, 19]. On the other hand, a hormonal connection between oxytocin and psychopathological clinical pictures has been discussed in a few studies. It was found that plasma oxytocin levels were higher in patients with bipolar 2 disorder during the depressive episode than in patients with major depression, which was actually supported by a molecular-biological study [20]. After treatment, the oxytocin level of bipolar 2 patients increased significantly, whereas the level decreased slightly in patients with major depression [21]. In a previous study, outpatients with major depression (*n* = 11) were found to have increased oxytocin levels compared to a healthy control group (*n* = 19) [22]. Interestingly, Cyranowski et al. [23] found that depressed women were more likely than controls to show a dysregulated pattern of peripheral oxytocin release. Animal studies regarding the neuronally-mediated, alcohol consumption-reducing effect of oxytocin are also particularly interesting [24]. Current reviews [25, 26] stated out that oxytocin affects alcohol consumption, but there are a need for more data concerning the other aspects of alcohol addiction.

As further rationale it has to mention that guilt relies on empathic concern and empathic concern is linked to interpersonal attachment, hence relating directly to oxytocin.

This explorative pilot study aimed to investigate the following aspects in more detail: Is there a difference in the degree of shame and guilt in patients with depressive disorder and in patients with alcohol addiction compared to healthy volunteers, and do biographical, clinical, or psychometric parameters influence the outcome of shame and guilt? Is there any correlation between personality and guilt proneness and/or shame proneness? Finally, another goal was to assess if patients suffering from two different mental disorders compared to healthy controls differed in their oxytocin levels in relationship to guilt and shame proneness.

## Methods

### Participants

A total of 85 participants (41 women and 44 men) were examined in this study. Of these, 31 patients fulfilled the criteria of depression (ICD-10: F32.X/F33.X (mean age: 35.2 (SD=10.7) years)), 23 patients (mean age: 52.1 (SD=9.0) years) had alcohol addiction according to ICD-10 (F10.2) and 31 subjects formed the healthy control group (mean age: 39.1 (SD=15.0) years). A detailed description of the three groups can be found in Table 1.

**Table 1:** Sociodemographic characteristics

Parameter	Healthy volunteers *N* = 31	Alcohol dependent patients *N* = 23	Depressive patients *N* = 31	*p*-value*
**Age**, mean (SD),	39,1 (15.0)	52.1 (9.0)	35.2 (10.7)	*p* ≤ 0.001[3]
Range (Years)	18–66	31–67	20–57	
**Gender**				n. s
Female, n	17 (54.8%)	7 (30.4%)	17 (54.8%)	
Male, n	14 (45.2%)	16 (69.6%)	14(45.2%)	
**Marital status**				*p* = 0.001
Single, n	20 (64.5%)	3 (13%)	17 (54.8%)	
married, n	10 (32.3%)	13 (56.5%)	13 (41.9%)	
divorced, n	0	6 (26.1%)	1 (3.2%)	
widowed, n	1 (3.2%)	1 (4.3%)	0	
Current Partnership				n. s
-Yes	25 (80.6%)	15 (65.2%)	20 64.5%)	
-No	6 (19.4%)	8 (34.8%)	11 (35.5%)	
**Graduation**				*p* = 0.002
High school, n	25 (80.6%)	6 (26.1%)	21 (67.7%)	
Junior high school, n	6 (19.4%)	10 (43.5%)	7 (22.6%)	
Low school., n	0	6 (26.1%)	3 (9.7%)	
No, n	0	1 (4.3%)	0	
**Occupational status**				*p* ≤ 0.001
Current employment (Including be a student), n	29 (93.5%)	10 (43.5%)	25 (80.6%)	
No current Job, n	1 (3.25%)	11 (47.8%)	6(19.4%)	
Retirement	1 (3.25%)	2 (8.7%)	0	
** Having Children**	20 (64.5%)	14 (60.9%)	7 (22.6%)	*p* = 0.032
** Experience of neglect by own parents,** n	0	4 (17.4%)	8 (25.8%)	*p* = 0.012

*SD* Standard deviation

* x[2] (Pearson *p*-value), [3] ANOVA: F _2/97_ = 8.458

All psychiatric patients were recruited and examined during their treatment at the wards of the Department of Psychiatry (LWL University Hospital of Medical Faculty of the Ruhr-University Bochum). The examination of the healthy volunteers also took place at the LWL University Hospital Bochum. Recruitment was done via notices and flyers. All participants have signed their informed consent.

Patients and healthy volunteers aged between 18 and 67 years were included. Further inclusion criteria were a verbal IQ > 70, sufficient German language skills, ability to give consent and consent to participate in the study in accordance with the Helsinki Declaration and ICH-GCP requirements. Exclusion criteria of the study were severe somatic illness as well as other mental disorders (including intellectual disabilities (ICD10: F70.−0.70.9), schizophrenia (ICD10: F20.-. F20.9), or brain-organic disorders (ICD10 F.06-F.06–9, addiction on illegal drugs), acute suicidal tendencies or behavior endangering others, as well as lack of consent to participate in the study. In the group of depressive patients, addiction (ICD10: F10.-. F19) was also an exclusion criterion.

The use of corticosteroids and sex hormones, except for oral contraceptives, also led to exclusion from the study due to a possible influence on the oxytocin level. In the alcohol-dependent patients, a depressive concomitant disease was not an exclusion criterion; only 5 (21.7%) of these patients had a depressive comorbidity.

Furthermore, psychopharmacotherapy of depressive patients and patients with alcohol addiction was also not an exclusion criterion. In this respect, 64.5% of the patients with a depressive illness (*n* = 20) received monotherapy, whereby antidepressants from the SSRI group (e.g., sertraline, escitalopram, paroxetine) were predominantly used (*n* = 13). Furthermore, 6 of the depressed patients received combination treatment (second antidepressant or a sedating antipsychotic medication e.g., promethazine, quetiapine) and 5 patients with depression had no psychopharmacotherapy at the time of study inclusion. The patients with alcohol addiction all received a magnesium preparation and vitamin B substitution. In addition, 15 of the 23 patients (65.2%) received at least one psychotropic drug (e.g., gabapentin, levetiracetam, doxepin) and 8 of them a combination treatment for the additional control of inner restlessness (e.g., pipamperone, promethazine). Since the alcohol-dependent patients were only examined at the end of withdrawal treatment, none of them took a benzodiazepine or clomethiazole. A detailed anamnesis was taken from all depressive and alcohol dependent patients and healthy volunteers in a semi-structured interview (duration 45–60 min). The psychometric characteristics including the shame and guilty proneness was gathered using various questionnaires. The study was approved by the local ethics committee (No.: 19–6733) of the Medical Faculty of the Ruhr-University Bochum.

## Oxytocin analysis

Peripheral venous blood was collected from each subject between 8:00 and 9:00 a.m. after eight hours of overnight fasting before the intake of any food or medication. Blood was sampled in EDTA Vacutainer tubes with a coagulation activator and centrifuged at 1300 RCF at 4 °C for 10 min. No more than 20 min elapsed between blood collection and centrifugation. After centrifugation, exactly 0.5 ml of plasma was pipetted into 2 ml Eppendorf tubes (Eppendorf AG 0030 120.094; pipette used: Eppendorf Research Plus). Serum samples were stored at − 80 °C until they could be analyzed. The analysis of the blood samples was carried out in the RIAgnosis laboratory of Prof. Dr. Rainer Landgraf in Sinzing. The samples were transported on dry ice. To avoid inter-assay variability, all plasma samples were sent and analyzed at the same time. The laboratory RIAgnosis offers a radioimmunoassay as a method of analysis. An assay sensitivity in the range of 0.1 pg per sample was reported by the laboratory. The coefficient of variation (CV) was < 1.

## Measuring instruments

### Psychometric and personality and instruments

The Beck Depression Inventory (BDI-II [27, 28] was used to assess possible depressive symptoms in the subjects. The Clinical Impression Score (CGI) was used to measure the overall severity of the patients’ mental illness. The Personal and Social Performance Scale (PSP) is a valid, reliable, and standardized measuring instrument for recording psychosocial functional level [29, 30]. The NEO-Five Factor Inventory (FFI), according to Costa and McCrae [31] (German translation by Borkenau and Ostendorf, revised [32]), is used to measure the following five different personality traits: Neuroticism (N), Extraversion (E), Openness to experience (O), Agreeableness (A), and Conscientiousness (C). For the alcohol addiction patients, the Scale for the Assessment of the Severity of Alcohol Addiction (= SESA [33]) was additionally collected to assess alcohol consumption and the severity of the alcohol disease. This consists of a total of 28 items, which are divided into seven subscales (narrowing of the drinking repertoire, somatic withdrawal symptoms, alcohol consumption to avoid or alleviate withdrawal, craving, increase in tolerance, extreme increase in tolerance, and decrease in tolerance). A total of 18 items refer to the patient’s most recent drinking habits. These items can be scored between 0 and 4. Ten items refer to the patient's overall drinking history. These items can be answered by the patient with “yes” (corresponding to 1 point) or “no” (corresponding to 0 points). When evaluating the SESA questionnaire, summed values of the individual subscales as well as an overall test value can be determined.

## Guilt and shame questionnaires

The questionnaires that separately assess guilt (IGQ = Interpersonal Guilt Questionnaire; in the German version “FIS = Fragebogen zu interpersonellen Schuldgefühlen”) and shame (SHAME) are distinguished from the questionnaires that assess both at the same time (TOSCA and SCV): The use of the different questionnaires to capture feelings of guilt and shame allowed both different aspects and expressions of these emotions to be captured to the greatest extent possible as emotions during states, but not traits of individuals, and to identify the behavioral and cognitive aspects that are associated with and can accompany the response to these emotions.

The “Interpersonal Guilt Questionnaire” (IGQ [34]), which was further developed and published in the German version by Albani et al., [35] consists of 21 items assigned to the three subscales “Survivor Guilt”, “Separation Guilt”, and “Omnipotent Responsibility Guilt” (seven items per scale). The scale allows the formation of sum values of the individual subscales as well as an overall value, which can be seen as an expression of the extent of the interpersonal feeling of guilt (Maximal sum score: 105).

The questionnaire “Shame Assessment for Multifarious Expressions of shame” (SHAME) was developed by Scheel et al. [36] to assess positive and negative aspects of shame in a differentiated way. The questionnaire contains three scales (physical, cognitive, and existential shame) with seven items each. Cognitive shame arises when one’s own moral or mental values are violated, when one is socially excluded, or when one has a subjective feeling of incompetence. Physical shame includes the areas of sexuality, intimacy, and the physical ideal image. Existential shame is a permanently experienced shame in the context of subjective self-esteem and self-confidence problems. In addition to the subscale sum value, whereby the higher the sum value, the higher the corresponding sense of shame, a total value can also be formed as a measure of the sense of shame [36, 37]. (Maximal sum score: 105).

The Test of Self-Conscious Affects (TOSCA [38]; German version: Kocherscheidt et al. [39]) allows the recording of self-evaluating emotions such as guilt and shame, but also pride as well as externalization of responsibility and distancing in the sense of indifference. The questionnaire consists of short descriptions of 15 everyday situations from private and professional life, divided into five affect-positive and ten affect-negative scenarios. The reaction to each situation is indicated by the respondent on a five-point scale (from 1 = does not apply to 5 = applies completely). The TOSCA allows a differentiated statement between guilt and shame and their expression (by forming the sum values by adding the corresponding individual items), considering affective, cognitive, and behavioral aspects [39]. (Maximal sum score: 75).

The Shame-Guilt Scale (SCV = Scala di Colpa e Vergogna) by Battacchi et al., [40, 41] published in the German version by Suslow et al., [7] is a self-rating questionnaire consisting of 11 scales and a total of 45 items. The scale was developed to measure several aspects of proneness to shame and guilt. In addition to recording various aspects that are associated with a tendency to feelings of guilt and shame, the scale also allows a distinction to be made between adaptive (functional) and maladaptive dysfunctional guilt and shame susceptibility [7]. Five subscales record shame-relevant aspects such as “Self-blame for one’s limits” (five items), “Shyness” (five items), “Social shame anxiety” (four items), “Self-blame for one’s mental contents” (three items), and “Reserve” (“antidelophilia” (three items)). The guilt-related traits are assessed by means of four subscales such as “Punitive guilt” (three items), “Self-Blame for overt behavior (three items), “Moral shame” (three items), and “Empathy reparation” (three items). Two subscales (“Persecutory guilt” and “Fear of social rejection” with three items each) are used to capture the need for competitiveness and domination of others and the need for attention and approval from others. Each item can be answered on a numerical five-point scale from 0 = “Does not apply to me at all” to 4 = “Applies to me completely” [7]. (Maximal sum score: 45).

## Statistical analyses

The descriptive and inferential statistical calculations were performed with Statistical Package for the Social Sciences (SPSS 26.0, SPSS Inc., Chicago). Non-parametric tests were chosen for statistical analyses since data were not normally distributed according to the Kolmogorov–Smirnov-Test. The level of statistical significance was set to α = 0.05. To adjust for multiple comparisons, a Bonferroni correction was partly applied for the group comparisons and the multiple linear regression analysis, resulting in a significance level at α = 0.001 in general.. Statistically significant group differences were determined using non-parametric tests (Mann–Whitney-U-tests, x[2]; ANOVA with multiple comparisons and post hoc tests),, while Spearman coefficients were calculated for correlative relationships. Finally, a stepwise multiple linear regression analysis concerning various factors possibly influencing the guilt and shame scales were conducted.

## Results

### Sociodemographic and clinical characteristics

The socio-demographic findings of the different samples are shown in Table 1.

Significant differences for age occurred as shown by the group effect in the ANOVA (F _2/97_ = 8.58, *p* ≤ 0.001). Although the group of depressive patients (DD) had a significantly younger age at first manifestation (mean: 27.2 (SD = 9.9)) as the patients with alcohol addiction (mean: 36.7 (SD = 12.3); *p* = 0.003), there was also a significant difference in the duration of the disease between the two patient groups (DD mean: 8.1 (SD = 9.8) years vs. AA mean: 15.5 (SD = 12.3) years, *p* = 0.017). A positive family history of mental illness in general was found in 58.1% of DD and in 26.1% of AA. Interestingly, 52.2% of the AA reported the presence of alcohol addiction in at least one of the parents; this was significantly less frequent in the depressed patients (22.7%). In both patient groups, 26% reported having experienced physical violence in their own childhood; among the healthy patients, the proportion was 6.5%.

It should be mentioned that the AA with an average SESA total value (60.0 (SD = 17.3), range 29.2–85.7) had a medium severity of alcohol addiction.

## Psychometric characteristics

Descriptive results for the psychometric findings (BDI-II, CGI, PSP) including the NEO-FFI personality dimensions (Big-Five) are described in Table 2. In the ANOVA with a normal distribution, significant differences were found regarding psychosocial limitations in the PSP (F2/82 = 577.099, *p* ≤ 0.001) and depressiveness measured by the BDI-II (F2/82 = 49.010, *p* ≤ 0.001). Post-hoc analyses (Games-Howell) showed significant differences between the patients with depressive disorders and the healthy controls in terms of depressiveness, which was highest in this group (*p* ≤ 0.001), and in psychosocial functioning (*p* ≤ 0.001). The patients with alcohol addiction also differed significantly in terms of depressiveness (*p* ≤ 0.001) from the healthy controls but also from the depressive patients (*p* = 0.013). Regarding the psychosocial functioning level, this was most impaired in the alcohol-dependent patients and differed significantly from the depressed patients (*p* = 0.005) and the healthy subjects (*p* ≤ 0.001).

**Table 2:** Psychometric characteristics including guilt and shame scales

Parameter	Healthy Controls *N* = 31	Patients with alcohol dependence *N* = 23	Patients with depression *N* = 31	ANOVA
PSP, M, (SD)	95 (0)	69.6 (3.0)	72.9 (4.4)	*p* ≤ 0.001
CGI, M, (SD)	1.0 (0)	5.0 (0)	4.6 (0.5)	*p* ≤ 0.001
BDI-II, M, (SD)	3.7 (4.1)	16.8 (10.3)	25.2 (10.4)	*p* ≤ 0.001
Neuroticism, M, (SD)	1.4 (0.8)	2.2 (0.7)	2.8 (0.7)	*p* ≤ 0.001
Extraversion, M, (SD)	2.4 (0.5)	2.3 (0.6)	1.7 (0.8)	*p* ≤ 0.001
Openness, M, (SD)	2.5 (0.5)	2.2 (0.5)	2.4 (0.7)	n. s
Agreeableness M, (SD)	2.8 (0.5)	2.6 (0.4)	2.6 (0.5)	n. s
Conscientiousness, M, (SD)	3.1 (0.6)	2.6 (0.6)	2.2 (0.7)	*p* ≤ 0.001
TOSCA-Guilt	3.9 (0.4)	3.6 (0.6)	4.0 (0.5)	*F*_*2/82*_ = *3.833* *p* = *0.026*
TOSCA-Shame	2.7 (0.6)	2.6 (0.8)	3.2 (0.7)	*F*_*2/82*_ = *7.617* *p* = *0.001*
TOSCA-Externalization	3.3 (0.6)	3.7 (0.9)	3.2 (0.8)	n. s
TOSCA-Detachment	2.9 (0.5)	3.1 (0.5)	2.8 (0.6)	*F*_*2/82*_ = *8.565* *p* ≤ *0.001*
TOSCA-α-pride	3.9 (0.6)	3.6 (0.8)	3.2 (0.9)	*F*_*2/82*_ = *6.491* *p* = *0.002*
TOSCA-β-pride	4.0 (0.6)	3.8 (0.7)	3.4 (0.9)	*F*_*2/82*_ = *5.634* *p* = *0.005*
SCV-Self-Blame for One´s Limits	0.5 (0.7)	1.4 (1.2)	2.2 (1.0)	*F*_*2/82*_ = *25.381* *p* ≤ *0.001*
SCV-Shyness	0.8 (0.8)	1.2 (1.3)	1.9 (1.3)	*F*_*2/82*_ = *6.912* *p* = *0.002*
SCV-Social Schame Anxiety	1.1 (0.7)	1.5 (0.9)	2.1 (1.0)	*F*_*2/82*_ = *11.737* *p* ≤ *0.001*
SCV-Reserve	1.8 (0.7)	1.6 (1.0)	2.0 (1.1)	n. s
SCV-Self-Blame for One´s mental contents	0.7 (0.6)	1.5 (1.1)	1.7 (1.1)	*F*_*2/82*_ = *10.904* *p* ≤ *0.001*
SCV-Punitive Guilt	0.6 (0.8)	1.3 (1.3)	1.8 (1.1)	*F*_*2/82*_ = *10.002* *p* ≤ *0.001*
SCV-Self-Blame for Overt Behaviour	1.8 (1.1)	2.3 (1.3)	2.2 (1.2)	n. s
SCV-Moral Shame	2.1 (0.8)	2.2 (0.8)	2.0 (0.8)	n. s
SCV-Empathic-reparatory guilt	2.7 (0.8)	2.6 (0.9)	2.6 (0.8)	n. s
SCV-Persecutory Guilt	1.2 (0.7)	1.4 (0.8)	1.6 (0.9)	n. s
SCV-Fear of social rejection	0.4 (0.4)	0.5 (0.6)	0.6 (0.8)	n. s

*BDI-II* Beck-Depression-Inventory, *CGI* Clinical Global Impressions, *M* Mean value, *PSP* Personal and Social Performance Scale, *SCV* Shame-Guilt-Scale, *SD* Standard deviation, *TOSCA* Test of Self-Conscious Affects # statistic tendency

Regarding the Big Five dimensions, significant group differences were found in neuroticism (F2/82 = 28.791, *p* ≤ 0.001), extraversion (F2/82 = 10.398, *p* ≤ 0.001) and conscientiousness (F2/82 = 16.586, *p* ≤ 0.001). Post-hoc tests showed that the group of depressed patients had a significantly higher neuroticism score compared to the healthy subjects (*p* ≤ 0.001) and compared to the patients with alcohol addiction (*p* = 0.004). Patients with alcohol addiction also differed significantly (*p* = 0.003) from the healthy subjects, who were the least neurotic. In the case of extraversion, the depressive patients achieved the lowest values compared to both the group of alcoholics (*p* = 0.007) and the healthy subjects (*p* ≤ 0.001). Regarding conscientiousness, a significantly lower value was found for the depressive patients compared to the healthy test subjects (*p* ≤ 0.001), like the group of alcohol-dependent subjects (*p* = 0.007). Interestingly, the alcohol-dependent patients did not differ from the healthy volunteers regarding extraversion and conscientiousness.

### Guilt and shame findings

**Fig. 1:**
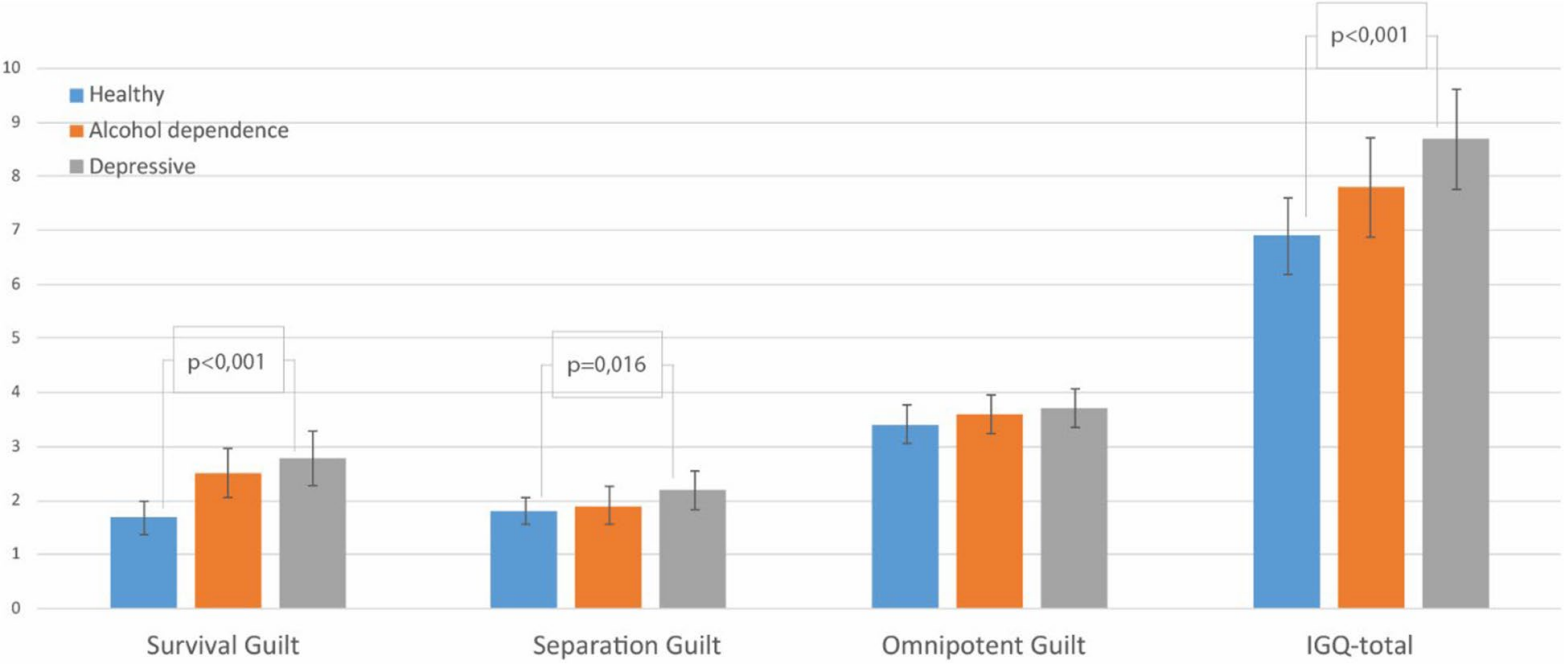
Group differences of Interpersonal Guilt Questionnaire (IGQ) (mean and standard error)

Significant differences for IGQ (Fig. 1) were found, as shown by the significant group effect in the ANOVA (IGQ-total score: F2/82 = 9.279, *p* ≤ 0.001), where the depressed patients showed the highest score for feelings of interpersonal guilt. They differed significantly from healthy controls (post-hoc-test: *p* ≤ 0.001), but not from alcohol-dependent patients. Univariate ANOVA of the IGQ subscales revealed significant group differences (Survival Guilt: (F2/82 = 9.279, *p* ≤ 0.001); these represent beliefs that achieving one’s own goals or personal success leads to the suffering of others) and for “Separation guilt” (F2/82 = 4.344, *p* = 0.016). Post-hoc tests showed that, for separation guilt feelings, which describe beliefs that separation or in addiction, for example in the form of detachment from parents, hurts close people, only the depressed patients differed significantly from the healthy subjects (post-hoc-test: *p* = 0.012). About feelings of survivor guilt, both patient groups differed significantly from the healthy subjects (post-hoc-test: DD: *p* ≤ 0.001 and AA: *p* = 0.020).

The comparison of proneness to shame measured by the SHAME questionnaire only showed a significant group difference in “bodily shame” (F2/82 = 5.991, *p* = 0.004), which was lowest in the patients with alcohol addiction (mean 1.5 (SD = 1.0)) and differed significantly from the highest bodily shame score of the depressive patients (mean 2.5 (SD = 1.2), *p* = 0.004).

Findings of the guilt and shame scales TOSCA and SCV are also shown in Table 2. Apart from the TOSCA externalization subscale, significant differences were found in all other TOSCA subscales. In the post-hoc tests, unfavorable, significant differences were found especially for the depressive patients regarding the expression of shame (compared to the healthy subjects (*p* = 0.004) and the alcohol-dependent patients (*p* = 0.007)) and distancing (compared to healthy subjects (*p* = 0.023) and alcohol-dependent patients (*p* = 0.001)). Also, for the depressive patients, significantly lower expression values of self-pride (*p* = 0.002) and behavioral pride (*p* = 0.005) were found, only in comparison with the healthy controls.

On the SCV, significant group differences were found in only five subscales (out of 11), as can be seen in Table 2. Here due to the post-hoc-tests, too, the depressed patients showed significantly elevated “Self-Blame for one’s limits” (*p* ≤ 0.001) and “Self-blame for one’s mental contents” (*p* ≤ 0.001), they were shyer (*p* = 0.001) with increased “Social shame anxiety” (*p* ≤ 0.001) and higher “Punitive guilt” (*p* ≤ 0.001) compared to the healthy controls. However, there were also a few significant differences for the patients with an alcohol addiction, on the one hand regarding “Self-blame for one’s limits” (compared to healthy *p* = 0.011 and depressive patients *p* = 0.019). On the other hand, AD patients differed on social shame anxiety compared to the depressive patients (*p* = 0.036) and on “Self-blame for one’s mental contents” compared to the healthy subjects (*p* = 0.010).

In the multivariate analyses carried out with the covariates age, education, neuroticism, and BDI-II, the group differences found could be confirmed for IGQ, TOSCA, SHAME, and SCV (F4/75 = 2.5 to 4.2; *p* ≤ 0.05), whereby the influence of BDI-II was strongest in these models.

To sum up, patients with DD showed a maladaptive guilt and shame profile, characterized by increased interpersonal feelings of guilt and increased proneness of shame. Patients with AA were characterized by the lowest reserve and antidelophilic attitude.

### Correlative relationships

Concerning the correlations of socio-demographic and clinical characteristics, including SESA and the guilt and shame findings, several correlations were found. These were mainly related to the age of the study participants, the presence of a positive family history of mental illness, a positive history of physical violence and neglect in childhood, and finally the duration of the illness and the age of onset (see Table 3). Further correlations between socio-demographic and clinical characteristics, including SESA and the guilt and shame findings, could not be ascertained.

**Table 3:** Correlations between biographic-clinical characteristics and Guilt and Shame Scalen

Variable	Age	Age of onset	Duration of mental illness	Positive family history	Experienceof physical violence	Parenteral Neglect
**IGQ**-total	*r* = −0.205 *p* = 0.060	*r* = 0.207 *p* = 0.057	***r* = *0.329*** ***p* = *0.002***	***r* = *0.234*** ***p* = *0.031***	***r* = *0.299*** ***p* = *0.005***	***r* = *0.410*** ***p* ≤ *0.001***
IGQ-survival	***r* =—*0.244*** ***p* = *0.024***	***r* =—*0.279*** ***p* = *0.010***	***r* = *0.459*** ***p* ≤ *0.001***	***r* = *0.294*** ***p* = *0.006***	***r* = *0.281*** ***p* = *0.009***	***r* = *0.417*** ***p* ≤ *0.001***
**SHAME**-total	***r* =—*0.245*** ***p* = *0.024***	* r* = −0.082 *p* = 0.455	*r* = 0.039 *p* = 0.722	*r* = 0.123 *p* = 0.264	*r* = 0.101 *p* = 0.360	*r* = 0.140 *p* = 0.202
SHAME-bodily	***r* =—*0.459*** ***p* ≤ *0.001***	*r* = −0.150 *p* = 0.171	*r* = −0.078 *p* = 0.479	*r* = 0.165 *p* = 0.131	*r* = 0.074 *p* = 0.503	*r* = 0.071 *p* = 0.518
**TOSCA**-Guilt	***r* =—*0.233*** ***p* = *0.032***	*r* = −0.146 *p* = 0.183	*r* = −0.139 *p* = 0.204	*r* = 0.207 *p* = 0.058	*r* = 0.150 *p* = 0.171	*r* = 0.060 *p* = 0.586
TOSCA-Shame	***r* =—*0.393*** ***p* ≤ *0.001***	*r* = −0.069 *p* = 0.531	*r* = 0.122 *p* = 0.266	***r* = *0.226*** ***p* = *0.038***	*r* = 0.121 *p* = 0.268	***r* = *0.217*** ***p* = *0.046***
TOSCA-DET	***r* = *0.265*** ***p* = *0.014***	*r* = −0.018 *p* = 0.874	*r* = −0.016 *p* = 0.888	***r* =—*0.305*** ***p* = *0.005***	*r* = −0.001 *p* = 0.996	*r* = −0.073 *p* = 0.505
TOSCA-EXT	***r* = *0.305*** ***p* = *0.005***	*r* = 0.115 *p* = 0.302	*r* = 0.115 *p* = 0.293	***r* =—*0.285*** ***p* = *0.008***	*r* = 0.185 *p* = 0.090	*r* = −0.051 *p* = 0.643
TOSCA– α-pride	*r* = 0.116 *p* = 0.291	*r* = −0.211 *p* = 0.052	***r* =—*0.243*** ***p* = *0.025***	*r* = −0.140 *p* = 0.201	*r* = 0.018 *p* = 0.867	***r* =—*0.337*** ***p* = *0.002***
**SCV**-SBL	***r* =—*0.283*** ***p* = *0.009***	***r* =—*0.282*** ***p* = *0.009***	***r* = *0.453*** ***p* ≤ *0.001***	*r* = 0.181 *p* = 0.098	***r* = *0.293*** ***p* = *0.006***	***r* = *0.319*** ***p* = *0.003***
SCV-Shyness	***r* =—*0.228*** ***p* = *0.036***	*r* = −0.002 *p* = 0.987	***r* = *0.274*** ***p* = *0.011***	*r* = 0.091 *p* = 0.408	*r* = 0.203 *p* = 0.062	*r* = 0.209 *p* = 0.055
SCV-SSA	***r* =—*0.236*** ***p* = *0.030***	*r* = 0.154 *p* = 0.159	***r* = *0.372*** ***p* ≤ *0.001***	*r* = 0.175 *p* = 0.108	***r* = *0.266*** ***p* = *0.014***	***r* = *0.220*** ***p* = *0.043***
SCV-SBM	*r* = −0.152 *p* = 0.166	*r* = 0.192 *p* = 0.078	***r* = *0.446*** ***p* ≤ *0.001***	*r* = 0.056 *p* = 0.614	***r* = *0.348*** ***p* = *0.001***	***r* = *0.284*** ***p* = *0.008***
SCV-PG	***r* =—*0.256*** ***p* = *0.018***	*r* = 0.160 *p* = 0.143	***r* = *0.336*** ***p* = *0.002***	*r* = 0.168 *p* = 0.125	***r* = *0.234*** ***p* = *0.031***	***r* = *0.258*** ***p* = *0.017***

*IGQ* Interpersonal Guilt Questionnaire, *SHAME* Shame assessment for Multifarious Expressions of shame, *SCV* Shame-Guilt-Scale, *SCV-SBL* Self-Blame for One´s Limits, *SCV-SSA* Social Shame Anxiety, *SCV-SBM* Self-Blame for One´s mental contents, *SCV-PG* Punitive Guilt, *TOSCA* Test of Self-Conscious Affects, *TOSCA-DET* Detachment, *TOSCA-EXT* Externalization

The most important correlations between the guilt and shame findings and the psychometric characteristics, such as the extent of depressiveness, the psychosocial functioning level, and the personality dimensions, are shown in Table 4.

**Table 4:** Correlations between Guilt and Shame scales with other psychometric characteristics

Variable	BDI-II	PSP	NEO-N	NEO-E	NEO-O	NEO-A	NEO-C
**IGQ**-Total	***r* = *0.607*** ***p* ≤ *0.001***	***r* = *−0.434*** ***p* ≤ *0.001***	***r* = *0.644*** ***p* ≤ *0.001***	***r* = *−0.423*** ***p* ≤ *0.001***	*r* = −0.046 *p* = 0.679	***r* = *−0.351*** ***p* = *0.001***	***r* = *−0.362*** ***p* = *0.001***
IGQ-Survival	***r* = *0.695*** ***p* ≤ *0.001***	***r* = *−0.525*** ***p* ≤ *0.001***	***r* = *0.729*** ***p* ≤ *0.001***	***r* = *−0.521*** ***p* ≤ *0.001***	*r* = −0.039 *p* = 0.722	***r* = *−0.478*** ***p* ≤ *0.001***	***r* = *−0.453*** ***p* ≤ *0.001***
IGQ-Separat	***r* = *0.361*** ***p* = *0.001***	***r* = *−0.256*** ***p* = *0.018***	***r* = *0.325*** ***p* = *0.002***	***r* = *−0.293*** ***p* = *0.007***	*r* = −0.138 *p* = 0.208	*r* = −0.161 *p* = 0.140	***r* = *−0.307*** ***p* = *0.004***
**SHAME**-total	***r* = *0.451*** ***p* ≤ *0.001***	*r* = −0.071 *p* = 0.519	***r* = *0.419*** ***p* ≤ *0.001***	***r* = *−0.551*** ***p* ≤ *0.001***	*r* = 0.109 *p* = 0.319	*r* = −0.200 *p* = 0.066	***r* = *−0.235*** ***p* = *0.031***
SHAME-bodily	***r* = *0.348*** ***p* = *0.001***	*r* = 0.029 *p* = 0.790	***r* = *0.391*** ***p* ≤ *0.001***	***r* = *−0.473*** ***p* ≤ *0.001***	*r* = 0.132 *p* = 0.230	*r* = −0.088 *p* = 0.421	*r* = −0.170 *p* = 0.120
SHAME-cognitive	***r* = *0.383*** ***p* ≤ *0.001***	*r* = −0.041 *p* = 0.711	***r* = *0.207*** ***p* = *0.006***	***r* = *−0.367*** ***p* = *0.001***	*r* = 0.058 *p* = 0.599	*r* = −0.111 *p* = 0.312	*r* = −0.130 *p* = 0.235
SHAME Existent	***r* = *0.343*** ***p* = *0.001***	***r* = *−0.215*** ***p* = *0.048***	***r* = *0.329*** ***p* = *0.002***	***r* = *−0.535*** ***p* ≤ *0.001***	r = 0.009 *p* = 0.936	***r* = *−0.283*** ***p* = *0.009***	*r* = −0.210 *p* = 0.054
**TOSCA**-Guilt	*r* = 0.165 *p* = 0.130	*r* = 0.116 *p* = 0.290	*r* = 0.180 *p* = 0.099	*r* = −0.128 *p* = 0.245	*r* = 0.102 *p* = 0.351	*r* = −0.021 *p* = 0.849	*r* = 0.126 *p* = 0.252
TOSCA-Shame	***r* = *0.561*** ***p* ≤ *0.001***	*r* = −0.138 *p* = 0.208	***r* = *0.606*** ***p* ≤ *0.001***	***r* = *−0.642*** ***p* ≤ *0.001***	*r* = 0.002 *p* = 0.989	***r* = *−0.390*** ***p* ≤ *0.001***	***r* = *−0.369*** ***p* = *0.001***
TOSCA-DET	***r* = *−0.243*** ***p* = *0.025***	*r* = 0.021 *p* = 0.850	***r* = *−0.323*** ***p* = *0.003***	***r* = *0.221*** ***p* = *0.042***	*r* = −0.170 *p* = 0.120	*r* = 0.153 *p* = 0.162	*r* = 0.031 *p* = 0.778
TOSCA-EXT	*r* = 0.076 *p* = 0.489	*r* = −0.089 *p* = 0.419	*r* = 0.104 *p* = 0.345	*r* = −0.094 *p* = 0.392	*r* = −0.174 *p* = 0.110	*r* = −0.171 *p* = 0.111	*r* = −0.121 *p* = 0.268
TOSCA- α-pride	***r* = *−0.299*** ***p* = *0.005***	***r* = *0.298*** ***p* = *0.006***	***r* = *−0.314*** ***p* = *0.003***	***r* = *0.270*** ***p* = *0.012***	*r* = −0.030 *p* = 0.783	*r* = 0.083 *p* = 0.449	***r* = *0.309*** ***p* = *0.004***
TOSCA ß-pride	***r* = *−0.214*** ***p* = *0.050***	r = 0.198 *p* = 0.069	***r* = *−0.246*** ***p* = *0.023***	***r* = *0.264*** ***p* = *0.015***	r = −0.049 *p* = 0.655	r = 0.040 *p* = 0.717	***r* = *0.294*** ***p* = *0.006***
**SCV**-SBL	***r* = *0.748*** ***p* ≤ *0.001***	***r* = *−0.462*** ***p* ≤ *0.001***	***r* = *0.815*** ***p* ≤ *0.001***	***r* = *−0.551*** ***p* ≤ *0.001***	*r* = −0.038 *p* = 0.729	***r* = *−0.325*** ***p* = *0.002***	***r* = *−0.648*** ***p* ≤ *0.001***
SCV-Shyness	***r* = *0.516*** ***p* ≤ *0.001***	***r* = *−0.230*** ***p* = *0.034***	***r* = *0.541*** ***p* ≤ *0.001***	***r* = *−0.753*** ***p* ≤ *0.001***	*r* = −0.001 *p* = 0.989	***r* = *−0.322*** ***p* = *0.003***	***r* = *−0.383*** ***p* ≤ *0.001***
SCV-SSA	***r* = *0.671*** ***p* ≤ *0.001***	***r* = *−0.357*** ***p* = *0.001***	***r* = *0.694*** ***p* ≤ *0.001***	***r* = *−0.613*** ***p* ≤ *0.001***	*r* = −0.041 *p* = 0.712	***r* = *−0.306*** ***p* = *0.004***	***r* = *−0.418*** ***p* ≤ *0.001***
SCV-SBM	***r* = *0.641*** ***p* ≤ *0.001***	***r* = *−0.420*** ***p* ≤ *0.001***	***r* = *0.646*** ***p* ≤ *0.001***	***r* = *−0.424*** ***p* ≤ *0.001***	*r* = −0.114 *p* = 0.300	***r* = *−0.438*** ***p* ≤ *0.001***	***r* = *−0.474*** ***p* ≤ *0.001***
SCV-PG	***r* = *0.634*** ***p* ≤ *0.001***	***r* = *−0.395*** ***p* ≤ *0.001***	***r* = *0.723*** ***p* ≤ *0.001***	***r* = *−0.542*** ***p* ≤ *0.001***	*r* = −0.052 *p* = 0.635	***r* = *−0.428*** ***p* ≤ *0.001***	***r* = *−0.409*** ***p* ≤ *0.001***

*IGQ* Interpersonal Guilt Questionnaire, *NEO-FFI* NEO-Five-Factor-Inventory, *N* Neuroticism, *E* Extraversion, *O* Openness, *A* Agreeableness, *C* Conscientiousness, *BDI-II* Beck-Depressions-Inventory, *SHAME* Shame assessment for Multifarious Expressions of shame, *SHAME-Existent* Existential, *SCV* Shame-Guilt-Scale, *SCV-SBL* Self-Blame for One´s Limits, *SCV-SSA* Social Shame Anxiety, *SCV-SBM* Self-Blame for One´s mental contents, *SCV-PG* Punitive Guilt, *TOSCA* Test of Self-Conscious Affects, *TOSCA-DET* Detachment, *TOSCA-EXT* Externalization, *PSP* Personal and Social Performance Scale

### Multiple linear regression analyses

Regarding the possible factors influencing the guilt and shame scales, stepwise multiple linear regression analyses were conducted. Here, the following factors were found to be particularly influential: age (β values ranging from −0.458 to −0.270; *p* ≤ 0.001 to *p* = 0.055) for “Bodily shame”, TOSCA-Shame, TOSCA-Externalization, and Moral shame (SCV); education (β = −0.280 to β =—0.212; *p* = 0.012 to *p* = 0. 031) for TOSCA Externalization, “Self-blame for overt behavior” and “fear of social rejection” (SCV); BDI -II (β = 0.663 to β = 0.331; *p* ≤ 0.001 to *p* = 0. 044) for IGQ-total and “survival guilt”, SHAME-total, SHAME-cognitive, SHAME-existential, TOSCA-Shame, and the SCV subscales “Self-Blame for one’s limits” and “Self-blame for one’s mental contents”, “Reserve”, “Punitive guilt”, and “Moral shame”; and finally, the factor neuroticism (β = 0.605 to β = 0.342; *p* < 0. 001 to *p* = 0.009) for IGQ total, IGQ-survival guilt, and the SCV subscales Self-blame for one’s limits”, “Self-blame for one’s mental contents”, and “Self-blame for overt behavior”, as well as “Punitive guilt”. In summary, age, education, depressiveness and neuroticism are the main regressors.

### Oxytocin analyses

With a mean value of 1.54 pg/ml (SD = 0.6), the oxytocin value was lowest in the patients with alcohol addiction compared to the healthy subjects (mean value 1.92 pg/ml (SD = 0.86)) and the depressive patients with a mean of 2.31 pg/ml (SD = 1.29). The significant group difference (F2/82 = 4.098, *p* = 0.020) resulted, as the post-hoc (Games-Howell) tests showed, only from the significantly different oxytocin levels between the two patient groups (*p* = 0.013).

For oxytocin, only two significant correlations were found in this study: on the one hand with gender (*r* =—0.256, *p* = 0.018) in the form that belonging to the female gender was associated with a higher oxytocin value, and on the other hand, there was an inverse correlation between the oxytocin value and the NEO-FFI dimension openness (r =—0261, *p* = 0.016).

Interestingly, no significant correlation coefficients were found between oxytocin and the guilt and shame scales. Thus, we can summarize, that oxytocin values were lowest in the patients with AA compared to the two other groups, but not related to guilt and shame.

## Discussion

In our explorative study about proneness to guilt and shame, we were able to show that patients with a depressive disorder and patients with an alcohol dependency differ significantly in this respect compared to mentally healthy subjects, but it cannot be assumed that there is a general increase in the feeling of guilt and shame in both psychiatric patient groups. By using different guilt and shame questionnaires, we were able for the first time to examine the expression of guilt and shame in a differentiated manner in two common psychiatric disorders such as depression and alcohol addiction and thereby identify two different nosology-specific profiles. In summary, the group of depressive patients in our study showed a maladaptive guilt and shame profile characterized by an increased interpersonal feeling of guilt, especially in the form of having offended or injured others through “egocentric and autonomy-relevant behavior” combined with self-blame and a punitive-self-critical attitude towards oneself. The tendency toward shame was also increased in the depressed patients in the form of increased social shame, shyness, and bodily shame, which includes areas of the physical ideal image as well as intimacy and sexuality, compared to the alcohol-dependent patients and healthy subjects.

In contrast, the patients with an alcohol dependency did not show an increased sense of interpersonal guilt compared to the healthy volunteers and were characterized by the lowest reserve and antidelophilic attitude in the sense of an increased tendency to self-presentation. This would also fit that they were less ashamed and more inclined to distance themselves as an expression of increased indifference and externalization of responsibility. Concerning our findings on guilt and shame in depressed patients, it can be stated that these are consistent with those in the literature (see the meta-analysis by Gambin and Sharp [16]). This is not surprising if one follows the model of the “cognitive triad”, since according to this, the depressed patient is characterized by a negative view of their own person, the world, and the future [42]. These negative, distorted assumptions, which not infrequently circulate as stereotypical, automated thoughts, are also accompanied by a persistent belief of having made a mistake and an exaggerated sense of responsibility for others and their wrongdoings. This behavior, attached to negativity, self-criticism, and self-blame, causes a lack of self-esteem, and leads to a destructive rejection of oneself [10]. Through shame and closely related feelings of guilt, the depressed patient ultimately tries to maintain a certain level of self-esteem [43].

In this context, a recent study by Duan et al. [44] should be mentioned, which comparatively examined 76 remitted patients with major depressive disorder (MDD) and 44 healthy subjects regarding the tendency to blame. It was shown that, in the group of patients, there was an increased maladaptive tendency to apologize for one’s own misbehavior, associated with all self-blaming emotions including shame, feelings of guilt self-contempt as well as disgust and indignation with oneself. Also, the tendency to hide and create an inner distance from oneself was higher in remitted patients with MDD than in healthy subjects [44]. Studies from Asia, such as that by Yeo et al., [15] confirm that, in this culture, proneness to guilt is associated primarily in the context of performance failure. While the healthy subjects (*n* = 71) felt a stronger sense of personal control in the case of success than in the case of failure and blamed external factors in the case of failure, the opposite was the case for the 71 depressed patients. Interestingly, the patients not only felt responsible for their failures, but were also more inclined to attribute successes to external factors.

However, it must also be mentioned that the results on guilt and shame in depressive patients, even if psychopathological and psychotherapeutic explanatory models seem comprehensible, are in no way consistent. Scheel et al., [37] for example, using the SHAME questionnaire, which was also used in our study, could not find any significant difference in the expression of shame in 17 female patients with MDD compared to a healthy control group. Also, not every depressive patient generally suffers from feelings of guilt [45]; moreover, the few well-founded studies do not contribute much to making general statements, not only because of the methodological differences, such as the use of different guilt and shame questionnaires.

Another problem is that the theoretical constructs of guilt and shame are also complex and divergent and are difficult to transfer to a nosologically inhomogeneous group such as depression. Our results regarding the expression of guilt and shame in alcohol-dependent patients are no less difficult to discuss in the context of the literature, since the overall number of studies is sparse, the results are inconsistent, and no differentiated study has been conducted to date. For example, earlier studies such as that by Meehan et al. [46] found an increased tendency to externalization and shame in all substance-dependent patients after a relapse, but a reduced tendency to feelings of guilt. Quiles et al. [47] and Dearing et al., [48] who conducted their studies mainly with students, but also with prison inmates with and without problematic substance use, could not find any difference concerning proneness to guilt and no close connection between guilt and substance abuse (in contrast to shame). Treeby et al., [49] on the other hand, sees a stronger link between guilt and alcohol abuse, especially since they were able to show in their studies that the subjects with an increased tendency to guilt used more strategies to avoid alcohol consumption. Shame had no effect in this respect. In this context, the work of Bilevicius et al. [50] should also be mentioned, who were able to show that shame is associated with addictive behavior such as pathological alcohol consumption or pathological gambling in connection with an additional depression or in the presence of vulnerable narcissism.

One of the few studies that examined clinically diagnosed patients with alcohol addiction is that by Grynberg et al. [13]. The comparison of 25 alcohol-dependent patients with 25 healthy controls matched according to age, gender, and education showed a significantly increased expression of guilt by means of the TOSCA questionnaire, while the groups did not differ from each other concerning proneness to shame [13]. In our study, the alcohol-dependent patients had the lowest guilt and shame scores in the TOSCA questionnaire and did not differ from the healthy subjects in the IGQ (interpersonal sense of guilt). In relation to shame, the AA patients showed the significantly lowest bodily shame. Several explanations could be used for the different results, such as the use of different questionnaires, different study populations with a predominance of female study participants, the extent of depressiveness, or personality dimensions.

Interestingly, the severity of alcohol addiction assessed by SESA did not play a significant role in the expression of guilt and shame in our study, like the work of Grynberg et al., [13] who also could not show a correlation between alcohol-specific characteristics and the guilt and shame profile. Despite the different findings regarding the extent of guilt and shame tendency, there is consensus that the guilt and shame profile of alcohol-dependent patients is also maladaptive. Unlike depressive patients, who, due to their altruism direct maladaptive shame, and guilt towards themselves and not towards others, thereby harming themselves [51], the dysfunctionality in alcohol-dependent patients, according to our findings, is that they are indifferent towards themselves and others and transfer responsibility to others (externalization) with less shame and thus consequently less motivation to change or make amends. This in turn often leads to a self-damaging vicious circle with fatal consequences for both the addicted patient and his/her relatives.

However, our findings also show that the proneness of guilt and proneness of shame not only occur as context-bound emotions, but, as already formulated early on by Lewis in 1971, are also closely related to personality traits, such as the “Big Five” dimensions here. Neuroticism, which is also considered a vulnerability factor for the development of psychiatric disorders, seems to favor the tendency towards increased guilt and shame, while dimensions such as extraversion and conscientiousness tend to have a protective effect and promote self-pride [52]. The association found in our study between reduced feelings of guilt and shame with increasing age is less surprising, especially since it is assumed that there is a functional perception and attitude towards guilt and shame due to age-related personality maturation and more self-reflection [37]. Of particular interest appear to be the correlations of interpersonal feelings of guilt and biographical-nosological characteristics such as a positive family history and experience of physical violence and neglect in childhood, which were found for the first time in our study. These characteristics were also clearly associated with increased social shame, anxiety, and increased self-blame for one’s mental contents.

This form of self-criticism and self-blame was similar in DD and AA patients and clearly more prevalent than in the healthy subjects; it can be seen as an indication that the patients are aware of their psychological condition and instability. It can be speculated to what extent our findings could be seen as an expression of feelings of guilt and shame in the context of early childhood trauma and mental illness because of insufficient coping with these. In this context, depressive patients try to control this through maladaptive “self-flagellation”, while patients with alcohol addiction try to suppress this through outwardly misdirected defense mechanisms.

The blood levels of oxytocin we found were lower in AA patients than in the healthy controls and especially depressive patients. We saw no relationship between oxytocin concentrations and the clinical features of alcohol addiction and depression. Oxytocin has been suggested to play an important role in the development of depression [21, 53–55]. Moreover, it has long been known that oxytocin could play a role in alcoholism and other drug-related behaviors [56, 57]. However, the connections between oxytocin and alcohol as well as depression have been studied so little and with mixed results. Lenz et al. [58] found significantly elevated oxytocin levels in patients with AA (in early abstinence from alcohol), more so in men than in women. However, Roschina et al. [59] reported lower serum levels of oxytocin (and β-endorphin) in patients with AA, particularly in those with a depressive comorbidity. Similar to our study, they also found no correlations between oxytocin and clinical as well as psychometric measures. One explanation could be that patients with alcohol addiction have lowered steroid levels which offer only few impulses for the oxytocin system.

### Limitations

Our descriptive study has several limitations. The first limitation is the small and inhomogeneous group population. Due to the exclusion criteria of severe organic disorders, patients with prolonged and strong alcohol addiction were excluded from the study population, which could have been a bias concerning the findings. This and the monocentric study design hardly allow any generally valid statements regarding the complex relationships between guilt and shame in the context of psychiatric illnesses. In addition, simultaneous presence of depressed mood was not an exclusion criterium in the alcohol patients. Moreover, we only measured serum levels of oxytocin and cannot be certain whether these correlate with effects as a neuroregulatory entity within the brain. In addition, the difference between AA and DD patients concerning oxytocin could be related to the usage of antidepressive medication in the latter group. Although the questionnaires used here have been validated, they are not routinely used and generally established instruments for examining guilt and shame in everyday clinical psychiatric practice.

## Conclusion

The proneness to maladaptive guilt and shame of various mental disorders appears to be variable and less dependent on specific disease aspects than on individual characteristics. In future, one might speculate that oxytocin could be used as treatment by nasal application to improve guilt and shame especially in AA patients to better stabilize them. Guilt and shame are important emotions and emotional mechanism in depression and alcohol addiction which should be stronger considered as before to break through basal processes of these two disorders. This should be broader investigated in further studies. Further multidimensional studies on guilt and shame in much larger patient groups could contribute to more well-founded differential findings and be implemented even more strongly in individualized psychotherapy. In terms of clinical implication of the above results, it is important to underline that working with shame and guilt is crucial, but can be hard for the patients and also for the therapists as well, depending on their emotional reaction elicited in the relationship [60].

## Data Availability

The datasets used and/or analysed during the current study available from the corresponding author on reasonable request.

## Abbreviations

AA: Alcohol addiction
BDI: Beck Depression Inventory
CGI: Clinical Impression Score
DD: Depressive disorder
ICD: International Classification of Diseases
IGQ: Interpersonal Guilt Questionnaire
FFI: NEO-Five Factor Inventory
PSP: Personal and Social Performance Scale
SCV: Shame-Guilt Scale (Scala di Colpa e Vergogna)
SESA: Scale for the Assessment of the Severity of Alcohol Addiction
SHAME: Shame Assessment for Multifarious Expressions of shame
TOSCA: Test of Self-Conscious Affects
